# The diagnosis that should speak its name: why it is ethically right to diagnose and treat personality disorder during adolescence

**DOI:** 10.3389/fpsyt.2023.1130417

**Published:** 2023-05-09

**Authors:** Joost Hutsebaut, Sharon L. Clarke, Andrew M. Chanen

**Affiliations:** ^1^Department of Medical and Clinical Psychology, Tilburg University, Tilburg, Netherlands; ^2^Viersprong Institute for the Study on Personality Disorders, Halsteren, Netherlands; ^3^Orygen, Melbourne, VIC, Australia; ^4^Centre for Youth Mental Health, The University of Melbourne, Parkville, VIC, Australia

**Keywords:** personality disorder, psychopathology, adolescents, prevention, early detection, early intervention, staged care, stigma

## Abstract

Although national guidelines explicitly state that personality disorder can be diagnosed and treated in young people aged 12 to 18 years (adolescents), most clinicians remain hesitant. This creates a gap between science and practice, which we argue is largely motivated by moral reasons and, therefore, is best challenged by ethical arguments. We provide seven arguments in support of the notion that it is ethically right to diagnose and treat personality disorder when it occurs in adolescents. Central to these arguments is the scientific evidence that features of personality disorder are among the best predictors of a complex cluster of psychopathology leading to impairments in many areas of current and future mental, social and vocational functioning. We argue that intervention during adolescence and young adulthood is not only humane, but also critical for efforts to avert the longstanding psychosocial and health problems that seem refractory to treatment in adults with personality disorder. Moreover, we argue that regular services are often inadequately equipped to meet the needs of young people with personality disorder and that the common ‘stepped-care’ approach should be replaced by a ‘staged-care’ approach. Finally, we argue that early detection and intervention might have anti-stigmatizing effects, similar to other areas of healthcare in which stigmatizing labels have changed meaning when the conditions to which they refer have become more amenable to treatment.

## 1. Introduction

More than a decade ago, Chanen and McCutcheon (1) published a paper with the telling title “The diagnosis that dare not speak its name.” This title alluded to the prevailing taboo against the diagnosis of a personality disorder (PD) in adolescents. Some years later, that taboo was documented in a survey study by Laurenssen et al. (2), showing that only 8.7% of 566 psychologists surveyed actually made the diagnosis of a PD in adolescents, even though 57.8% indicated that they recognized this disorder among the adolescents they treated. Hesitation (or even reluctance) to make the diagnosis was mainly informed by the beliefs that features of PD are transient in adolescence (41.2%) and/or that PD diagnoses were not allowed by DSM-IV-TR (25.9%). Psychologists working in primary and secondary care avoided the diagnosis more than those working in more specialized settings.

Over the past 15 years, there has been a steep increase in empirical studies in this field. Several review papers have been published [e.g., (3–6)] supporting the reliability and validity of the diagnosis of PD in adolescents. National guidelines for the assessment and treatment of borderline PD (BPD) have explicitly addressed the issue [e.g., (7, 8)]. Although there is international consensus among experts in the field that PD can and should be diagnosed in adolescence (9), it remains to be seen whether this will lead to cultural change in mainstream clinical practice. The title of Chanen and McCutcheon’s paper captured the emotional tone of clinicians’ concerns, suggesting anxiety or fear, and its counterpoint, hostility. We believe that this primarily relates to, and finds its expression in, moral or ethical concerns about the harms of making a PD diagnosis at young age (10). Such a motivation might be more powerful than any purely rational, scientific argument and jeopardizes the prospect that the scientific progress of the last 15 years might produce any change in the culture of clinical services. Consequently, this paper takes a different approach, presenting a narrative review of research data that suggests that early diagnosis and treatment of PD is just and ethical. We build our case around seven arguments. While most data are based upon studies of BPD, there is both evidence and consensus that BPD is representative of generic personality pathology, so findings are generalizable to other types of PD (11).

## 2. Seven arguments why early diagnosis and treatment of PD is just and ethical

### 2.1. Prevention and early intervention are common strategies in health care

Virtually every discipline within somatic and mental health care has strategies for early detection of progressive, harmful and/or potentially life-threatening conditions. The reason is clear and simple: survival rates increase significantly when serious conditions are detected early. Moreover, treatment is usually less invasive and causes fewer adverse effects in earlier stages of the disease. The field of oncology provides paradigmatic examples of this, such as in breast cancer. International guidelines recommend population-based detection strategies in early stages of disease and rapid implementation of evidence-based interventions aimed at cure, or, at least, stopping disease progression (12). Such programs have been proven to be cost-effective (13). Survival rates for different types of cancer have improved because of early detection and intervention strategies (14).

These ideas have also gained acceptance in the field of mental health care (15), especially in the field of early intervention for psychosis [e.g., (16)]. Various studies have demonstrated the effectiveness and cost-effectiveness of strategies for early detection and intervention in the treatment of psychotic disorders [e.g., (17)]. Following on from this idea, clinical staging (analogous to cancer staging) has been developed to encompass a wide range of mental disorders (18, 19). A crucial parameter within this approach is the ‘duration of untreated illness’, i.e., the time between the onset of symptoms and the start of (appropriate) treatment. Studies have repeatedly shown that duration of untreated illness predicts disease outcome for depressive (20), anxiety (21), obsessive–compulsive (22) and psychotic (23) disorders.

Taken together, there are compelling arguments for the potential benefits of early detection and intervention strategies for all severe mental illness in young people (24). While the field of early intervention for severe mental illness remains contested by a small minority, excluding a severe mental disorder, such as PD, from such strategies is scientifically unjustified and would be discriminatory.

### 2.2. Features of borderline PD are robust markers of severity of present psychopathology

One of the basic prerequisites for preventive detection aimed at early intervention is that the condition being detected causes important health problems (25). For example, detecting BPD in its early stages might only be useful when these features refer to manifestations of severe psychopathology. There is still a popular belief among some clinicians in many countries that, “every young person is a bit borderline.” This notion is based upon the idea that the typical features of BPD -such as emotional instability, impulsivity, fluctuations in self-esteem, and identity disturbance – do not differ from normal developmental phenomena during puberty and, therefore, might not be indicative of psychopathology. More than two decades of research has thoroughly refuted this popular belief (26).

Firstly, although BPD features clearly increase during puberty and young adulthood (27), the prevalence of PD among adolescents largely resembles prevalence estimates among samples of adults (28–30) and is well below rates that might be expected if PD criteria (even partly) captured normative developmental phenomena. Secondly, PD criteria in adolescents independently predict a broad array of associated current problems in mental, social, and academic functioning. Moreover, these PD criteria are more predictive of such problems than classic symptoms of frequently occurring mental state (‘Axis 1’) disorders, such as mood, anxiety or conduct disorders, suggesting that PD criteria are highly informative markers for severe psychopathology. For examples, short-term correlates of PD in adolescents include increased suicidality, school dropout, risk of substance abuse, and increased use of health care services (31). About 60% of young people with BPD report suicidal thoughts, while 50–60% show self-injurious behavior (32). Burden of disease and health costs exceed those of adults with PD (33). Compared with adolescents with mental state disorders, adolescents with PD are more likely to have problems at school and fewer friends (34), more behavioral problems and difficulties at school (35), more alcohol abuse, drug use and nicotine dependence (36, 37), more sexual partners and unsafe sexual behavior (38, 39), riskier attitudes and norms toward sexual behavior (40), and more crisis admissions and medication use (41). Parents of young people with BPD report high levels of burden and parental stress (42). Families and carers of young people with BPD experience higher levels of negative experiences related to their role, compared, with caregivers of young people with first-episode psychosis (43). Thirdly, there is evidence that age of onset of BPD symptoms predicts developmental outcomes. A recent study demonstrated that earlier age of self-harm onset and longer duration of self-harm were both associated with increased frequency of subsequent periods of self-harm and risk of first suicide attempt (44). Lower age of onset was also associated with more repeated suicide attempts.

Taken together, these data strongly suggest that early emergence of typical (B)PD features is far from innocuous. Rather, the scientific evidence leads to the striking conclusion that features of (B)PD are robust markers of present problems and identify a group of young people at high risk for a broad range of adverse immediate outcomes. In fact, PD features appear to be more strongly associated with other problems areas, like academic and social functioning and risky health behavior, than are the classic internalizing and externalizing symptoms.

### 2.3. Borderline PD features are robust markers for future problems

One of the main arguments against diagnosis that was given by the professionals who were surveyed by Laurenssen et al. (2) was that PD features are unstable and transient between adolescence and (young) adulthood. Indeed, two decades of research has found that PD features are only moderately stable among young people (45). For example, a recent follow-up study found that, a decade later, about half of the participants diagnosed with BPD at the age of 15 had relinquished their diagnosis (46). However, objecting to the diagnosis on the basis of instability overlooks two key points. First, substantial data demonstrates similar levels of (in)stability among adults diagnosed with (B)PD, especially regarding the acute symptoms of the disorder (47), reflecting that the disorder itself is less durable than was previously assumed. Second, a narrow focus, solely upon prevention of personality disorders in adulthood, ignores the broad scope of future problems associated with PD diagnosis in adolescence, embodying the developmental psychopathology principle of ‘multifinality’.

Prospective and retrospective studies of young people with PD demonstrate that long term sequelae are much more widespread than the mere risk of developing adult PD. Poor outcomes in (young) adulthood include academic failure (35), poor vocational outcomes (48), poor physical health (49), excess mortality from medical conditions (50), increased suicide risk (51) and long term mental health problems and need for treatment (52, 53). Evidence clearly suggests that these poor outcomes are already established in the transition from adolescence to young adulthood in young persons with BPD (31, 48).

Consequent upon these data, a key preventive aim for early detection and treatment of PD in young people is prevention of the severe impairments in health, social, and vocational outcomes. While not unimportant, preventing BPD in adulthood is a secondary concern.

### 2.4. Adolescence (and young adulthood) is a sensitive period for the development of chronic psychosocial disability

Although treatment studies in adult PD patients usually show beneficial effects on the features of PD and upon associated problems, such as depression (54, 55), it is less clear to what extent these treatments also help with psychosocial recovery (4). Longitudinal, observational studies suggest that durable functional improvements are much more difficult to achieve than symptomatic improvements (47). This observation is not unique to PD. For example, psychotic disorders also show a similar gap between symptoms and functioning (56). This relates to the notion of a critical period for preventing long-term psychosocial disability.

This notion of a ‘critical period’ for early intervention is well established in the field of psychosis, where research supports early intervention to shorten the duration of acute illness, promote recovery, and to prevent secondary adverse outcomes, along with ‘youth specific’ mental health services to meet the needs of this expanded age group (16, 57, 58). The developmental period spanning adolescence and young adulthood between the ages of 12 and 25 years is the key period for establishing the foundations of adult role functioning, in which young people need to build the necessary self-regulatory, relational, vocational, and other skills for adult role functioning. The onset of PD features during this period has the potential to disrupt these developmental processes, which are difficult to compensate for in later life. The abovementioned findings regarding long-term outcomes [e.g., (48)] strongly suggest that BPD during the transition to adulthood interferes extensively with development of adult role functioning. Consequently, these young adults are over-represented among the unemployed, welfare recipients, socially marginalized, and medically unwell (51, 59, 60).

Early detection and intervention of personality pathology appears to be time-sensitive, defining an ‘enriched’ risk group and offering an opportunity to prevent long-term psychosocial disability and potentially irreversible psychosocial disadvantage. Studies testing this hypothesis are crucial to determine whether, and what kinds of, interventions might be effective.

### 2.5. Regular treatment is often inaccessible or less effective for young persons with BPD

Early intervention for young people with PD faces greater challenges than for other mental disorders in young people, as young people with PD do not even have parity of access to existing services. Young people with PD usually do not enter services equipped to address their personality impairments, are often refused access to psychotherapy programs, and respond less well than those without PD to existing treatments.

While there is some regional variation, Laurenssen et al. (2) found that only 6.5% of Dutch psychologists provide PD-oriented treatment, with none of these clinicians working in front-line services. Australian data provide a similar picture, showing that less than 1% of young people in primary care services were diagnosed with BPD (features). These young people received a variety of treatments, none of them specifically designed for BPD, and often in a very low dosage, with a mean of 3.4 sessions (61).

Where psychotherapy is provided, young people with (B)PD appear to respond less well to conventional treatments used in primary care. In a prospective follow-up study of young people receiving Cognitive Behavioral Therapy (CBT) for depressive problems, the subgroup females who also had BPD did not benefit from this treatment. These young people are up to four times more likely to have recurrent depressive episodes than those without BPD (62). One important reason for this non-response might be the high dropout rate from these treatments, with about 40–50% of young people with BPD disengaging from treatment prematurely, despite their high levels of distress and suffering (63).

There are few specific studies addressing this dropout rate. However, the main reason adolescents discontinue treatment appears to be related to perceived breakdowns in the therapeutic relationship (64). Ruptures that remain unaddressed, and for which the young person is held responsible, precede drop-out in adolescent treatment. This is likely to be more common among young people with PD, because the relational dynamics associated with PD also apply to the therapeutic relationship. A distinguishing feature of PD-specific treatment programs is that they directly address and/or actively manage these relational dynamics. Indeed, this appears to be one of the most important common features in effective PD treatment (65).

The therapeutic relationship is one of the most important common factors determining treatment success in young people (66) and treatment might be especially successful when a practitioner succeeds in building a genuine and authentic relationship (67). Young people with characteristics of (B)PD are highly sensitive interpersonally and, therefore, also very susceptible to what happens in the therapeutic relationship (68). Young people with BPD tend to hyper-mentalize (69), and might quickly become wary of the intentions of others, especially the sincerity of others’ intentions, leading to therapeutic ruptures. This can occur especially when the practitioners are too rigid in their adherence to treatment protocols (68), which might explain in part the modest improvements in various studies, and specifically the high rate of treatment dropout. PD-specific treatment can provide a framework for dealing with these relational dynamics among young people and their families.

### 2.6. Inappropriate or ineffective treatment might cause iatrogenic harm

Professionals often follow a stepped-care logic, commencing with more accessible, low-dose, and generic treatments and then only scaling up to more intensive, complex, and/or specialized treatments when these first-line treatments fail. The tacit assumption is that it is better to see if the condition can be treated with a mild intervention and only scale up if it is insufficient. A variant of this is the assumption that it is better to treat the mental state disorder (e.g., depression) first, before focusing on the PD. However, the fundamental requirement to ‘fail’ a particular step, in order to progress to the next step that was manifestly needed when the young person first presented risks prolonging the period of untreated (or ineffectively treated) illness, potentially leading to worse outcomes.

There is evidence that too brief interventions for serious disorders have aversive effects. Data from the Australian Headspace program for 12-to 25-year-olds found that 40% of young people with BPD features showed no progress at all after six sessions, while another 40% deteriorated. Moreover, 57% of youth deteriorated in terms of social functioning and 69% for quality of life (61). These data support suggestions that commencing with treatment that is inadequately tailored to the nature of the problems might increase the likelihood of harmful effects (70). Furthermore, data from the Improving Access to Psychological Therapy (IAPT) program in the United Kingdom suggests that non-response to a prior treatment might affect chances of recovery in subsequent treatment (71).

A ‘one size fits all’, stepped care approach is potentially harmful by delaying access to the right level of care, prolonging untreated illness, and potentially leading to loss of hope and demoralization among young people, families, and friends. For this reason, the notion of ‘staged care’ was introduced (72), which involves matching of care to the clinical stage of illness and needs of the young person. In this system of care, patients do not have to ‘fail treatment’ in order to eventually get to the level of care that they clearly needed in the first instance.

In short, the assumptions underlying the way that care is accessed and organized for young people with PD are not evidence-based and have high potential to cause further harm. Treatment allocation should be based on clinical stage, which requires identifying PD characteristics early and systematically (73).

### 2.7. Early detection and intervention might have anti-stigmatizing side-effects

Reluctance to diagnose PD in young people is often related to a fear of stigmatizing the young person with the (B)PD label (10). PD is often triply stigmatizing by suggesting ‘troublesome’, ‘untreatability’, and/or ‘life-long burden’. Notably, these negative attitudes and beliefs are often deeply rooted in the clinical cultures of health services and openly expressed by clinicians (74).

History is replete with examples of diagnoses that were not named in the past because of fear of stigma. HIV/AIDS and cancer are well known in the modern era. Among other reasons, their historical associations with ‘incurability’ and or lifestyle played important roles. Advances in treatment have meant that survival rates have improved, and cure is possible for many of these conditions. One key reason underpinning this have been the stage at which these disorders are detected and treated. Early initiation of appropriate treatment has had a positive effect upon prognosis and spawned large-scale public health campaigns. In mental health care, the psychosis field has been at the vanguard of similar developments. Earlier detection and treatment of young people with high-risk profiles for psychosis can delay or even prevent the onset of positive and negative symptoms of psychosis, thereby reducing the association between psychotic symptoms and untreatability or inevitable decline. We contend that a major reason why BPD is associated with long-term suffering, frequent treatment courses, and irreversible social impairment is the fact that the disorder is often diagnosed so late (75). Paradoxically, delaying diagnosis amplifies its stigmatizing effect. Rather than avoiding the diagnosis, we argue that changing the clinical culture that permits such attitudes and beliefs might actually address the root cause of the problem.

The PD diagnosis also evokes associations with ‘difficult’ or ‘troublesome’ clients. One concern is that giving this diagnosis might lead to ‘self-stigma’, whereby the person adopts such self-defeating labels (76). A label of ‘borderline’ might adversely affect identity and self-image during a critical developmental phase for the development of these capacities. However, it has been shown that young people with BPD severely stigmatize themselves even before they receive the diagnosis of BPD (77). Moreover, evidence shows that when the diagnosis is done well and delivered in a sensitive manner, people find this helpful (78). Nonetheless, what is lacking in the stigma discussion are the perspectives of a range of ‘experts by experience’. More research is needed on the experience of receiving the diagnosis of (B)PD by young people and families, comparing young people who are diagnosed at an early stage and those who might be diagnosed later in the course of illness.

## 3. Conclusion

The field of personality disorder in young people is still relatively under-developed. Yet, despite compelling scientific evidence, change has been slow. The seven arguments advanced in this paper support the notion that is morally right and justifiable to detect PD and to intervene early. Briefly summarized, diagnosis is warranted because it helps to identify a group of young people with high (and specific) current needs and very high risk for developing a range of severe and enduring negative outcomes. Two decades of research has clearly demonstrated that features of (B)PD are more informative than many other symptoms for identifying such a high-risk group, not only during adolescence, but also in the transition to (young) adulthood, and not only for negative outcomes in terms of mental disorders, but also in terms of psychosocial functioning. Moreover, regular services might be inaccessible or might be insufficient for the needs of the young person, resulting in multiple failed treatments and loss of hope for the young person, their family, and for clinicians. Finally, we argue that early detection might provide more timely and effective intervention, thereby reducing stigma, as has been demonstrated in other areas of health care. Staged care requires availability of a range of responses to meet the *actual* needs of the young person, when they need them, including diagnosis-specific interventions. Finally, over and above the compelling scientific evidence, there is moral imperative to improve access to interventions that meet the clinical needs of these young people and their families, and to match these to clinical ‘stage’, in order to deliver socially just care to this marginalized, under-served, and at-risk group.

## Author contributions

JH made the first draft of the paper. SC assisted with literature search and writing. AC revised and edited the paper. All authors contributed to the article and approved the submitted version.

## Conflict of interest

The authors declare that the research was conducted in the absence of any commercial or financial relationships that could be construed as a potential conflict of interest.

## Publisher’s note

All claims expressed in this article are solely those of the authors and do not necessarily represent those of their affiliated organizations, or those of the publisher, the editors and the reviewers. Any product that may be evaluated in this article, or claim that may be made by its manufacturer, is not guaranteed or endorsed by the publisher.

## Abbreviations

PD: personality disorder
BPD: borderline PD
CBT: Cognitive behavioral therapy
